# Rapid on-site detection of echinococcosis and schistosomiasis based on RPA

**DOI:** 10.1590/0074-02760230244

**Published:** 2024-10-11

**Authors:** Lvbo Tian, Ying Shi, Yu Yang, Yuchen Wang

**Affiliations:** 1 Sichuan International Travel Health Care Center, Port Epidemic Disease Monitor Key Laboratory of Sichuan Province, Chengdu, Sichuan, China; 2 Shanghai Customs College, Department of Inspection and Quarantine, Shanghai, China

**Keywords:** echinococcosis, schistosomiasis, RPA, on-site identification

## Abstract

**BACKGROUND:**

Echinococcosis and schistosomiasis, caused by parasitic worms, pose significant threats to millions of people in the world. Rapid and effective pathogen detection and epidemic control by public health authorities are urgently needed.

**OBJECTIVES:**

In this study, we aimed to develop rapid on-site detection method to detect echinococcosis and schistosomiasis.

**METHODS:**

Recombinase polymerase amplification (RPA) was utilised to examine its efficacy of detection of echinococcosis and schistosomiasis.

**FINDINGS:**

The detection probes for RPA were created through comparing parasitic genomes from international genomic data and the sequences generated by our group. We established an optimised RPA on-site testing platform, which significantly reduces the detection time (less than 30 min) and simplifies the operation (free of expensive equipment) as compared to traditional polymerase chain reaction (PCR) method.

**MAIN CONCLUSIONS:**

This RPA detection platform in our study for identifying echinococcosis or schistosomiasis pathogens would be greatly applicable for epidemic investigation, border screening, and early clinical diagnosis.

Echinococcosis, also known as hydatid disease, is a chronic zoonotic parasitic infection caused by the larvae (hydatid cysts) of the *Echinococcus* tapeworm in both humans and animals.^1^ *Echinococcus granulosus (E. granulosus)* and *Echinococcus multilocularis (E. multilocularis)* infections are the most common parasitic diseases.^2^ The disease primarily affects the liver, followed by the lungs, although other organs can also be affected.^3^ Clinical manifestations vary depending on the location, size of the cyst, and presence of complications. Echinococcosis is widespread globally distributed in most pastoral and rangeland areas of the world, with highly endemic areas in the eastern part of the Mediterranean region, northern Africa, southern and eastern Europe, at the southern tip of South America, in Central Asia, Siberia, and western China.^1^

Schistosomiasis caused by parasitic flatworms (blood flukes) of the genus *Schistosoma*, is an acute and chronic zoonotic parasitic disease transmitted through contacting with contaminated water containing cercariae.^4^ Schistosomiasis can infect multiple organs, including the liver, intestine, bladder, and urethra, leading to symptoms such as fever, oedema, or coughing in infected individuals.^5^ Diagnosis of schistosomiasis is the microscopic detection of parasite eggs present in urine or stool.^6^ The disease is particularly prevalent in tropical and subtropical regions, especially endemic in low-income rural communities without access to potable water, proper sanitation, and adequate healthcare facilities.^7^ It is prevalent over 70 countries, with considerable morbidity in parts of the Middle East, South America, Southeast Asia and, particularly, in sub-Saharan Africa, affecting approximately 230 million people.^8^ It is estimated that at least 90% of those requiring treatment for schistosomiasis live in Africa.^9^

Echinococcosis and schistosomiasis pose significant threats to public health. These diseases are predominantly concentrated in economically underdeveloped and remote areas, posing substantial health risks to local residents.^10^ Effective responses to these diseases require rapid and accurate identification of the pathogens, followed by appropriate prevention and control measures.^11^ ^,^ ^12^ On-Site rapid diagnostic tests during disease outbreaks are crucial to save time and financial resources. Swift and proactive strategic actions taken at the outbreak site can enhance the efficiency of prevention and control efforts, allowing for proactive measures to curb the echinococcosis and schistosomiasis epidemic.^13^ ^,^ ^14^ Thus, rapid, sensitive, affordable, and user-friendly diagnostic technologies and methods have become urgent necessities in the diagnosis of echinococcosis and schistosomiasis.

Recombinase polymerase amplification (RPA) is a highly sensitive and selective isothermal amplification technique that utilises recombinase, single-strand binding protein, and DNA polymerase to amplify nucleic acids under isothermal conditions (usually at 37-42ºC), with minimal sample preparation and capable of amplifying as low as 1-10 DNA target copies in less than 20 min.^15^ Moreover, the RPA showed high specificity, sensitivity, detection efficiency and robust resistance to interference.^16^ It is suitable for rapid, rough sample testing under field conditions. Consequently, the compact size of the detection instrument allows for easy portability. The RPA platform, as a constant temperature nucleic acid amplification technique, enabling DNA denaturation at room temperature without the need for expensive thermal cycler devices.^17^

This study focuses on establishing rapid on-site nucleic acid detection platforms for echinococcosis and schistosomiasis based on RPA technology. The RPA on-site rapid detection method developed in this research is fast, sensitive, and accurate, capable of detecting echinococcosis or schistosomiasis separately. In addition, RPA on-site testing platform simplifies the operation (free of expensive equipment) as compared to traditional polymerase chain reaction (PCR) method. It is applicable for on-site epidemic investigation, border screening, and early clinical diagnosis.

## MATERIALS AND METHODS

*Reagents and instruments* - Fully automated nucleic acid extractor (KinFisher Flex, Thermo, USA); RAA-1620 instrument (Jiangsu Qitian Genetic Biotechnology Co., Ltd.); RAA detection kit (Jiangsu Qitian Genetic Biotechnology Co., Ltd.); Primers and probes were synthesised by Shanghai Sangon Biotech Co., Ltd.

*Probe and primer design* - Multiple *Echinococcus* cox1 sequences were retrieved from the NCBI database with the following GenBank accession numbers: MH686292.1, KX082662.1, GQ856692.1, GQ856693.1, GQ168812.1, and GQ168811.1.

Multiple Japanese schistosome sequences were retrieved from the NCBI database with the following GenBank accession numbers: CV758431.1, CV758432.1, CV758433.1, CV758434.1, CV758429.1, and CV758430.1.

*Nucleic acid extraction* - *Echinococcus* and Japanese schistosome samples were extracted using the QIAGEN tissue DNA extraction kit. Extracted DNA samples were aliquoted and stored at -80ºC.

*RPA basic amplification* - The RPA basic amplification kit (RAA-1620, Jiangsu Qitian Genetic Biotechnology Co., Ltd.) was used following the manufacturer’s instructions. Briefly, amplification reactions were firstly prepared in 47.5 μL reaction mixture: 17.5 μL H_2_O, 25 μL Reaction Buffer A, 1 μL DNA sample, and 2 μL each primer (Table I). The reaction mixture was then mixed with lyophilised powder with RPA reaction enzyme. 2.5 μL of 280 mM magnesium acetate solution was added to each reaction tube and mixed well. The reaction was conducted in 39ºC for 40 min. 50 μL of phenol/chloroform (1:1) was then added to each reaction tube followed by 1 min centrifuge at 12000 rpm. 10 μL of the upper layer solution was used for agarose gel electrophoresis (typically using a gel concentration of 1.5-2%) to obtain the test results.

**TABLE I:** *Echinococcus* and schistosome recombinase polymerase amplification (RPA) detection reaction system

Components	Volumes (μL)
Primer A (10 μM)	2
Primer B (10 μM)	2
Reaction buffer A	25
ddH2O	17.5
Template	1

*RPA fluorescent amplification* - RPA fluorescent amplification was performed using RPA fluorescent amplification kit (Jiangsu Qitian Genetic Biotechnology Co., Ltd.) according to the instructions. The 47.5 μL reaction mix contains 25 μL of reaction buffer, 2.1 μL each of forward and reverse primers (10 μM), 0.6 μL probe (10 μM), 1 μL template, and 16.5 μL double-distilled water. The reaction mix was then added with lyophilised powder with reaction enzyme, and 2.5 μL of 280 mM magnesium acetate solution was added. The final reaction mix was performed at 39ºC for 30 min in the RAA-F1620 instrument (Jiangsu Qitian Bio-Tech Co. Ltd., China).

*Sensitivity experiment* - Based on the optimised primer and probe sequences, the target region for amplification was determined. The corresponding plasmid fragments were synthesised as sensitivity test templates and diluted tenfold in DEPC water across seven gradients. RPA was used to test their sensitivity.

*Statistical analysis* - DNAman software 8.0 was used for alignment and design. GraphPad Prism software 9.0 was used to perform the graph of all the data.

## RESULTS AND DISCUSSION

**Fig. 1:**
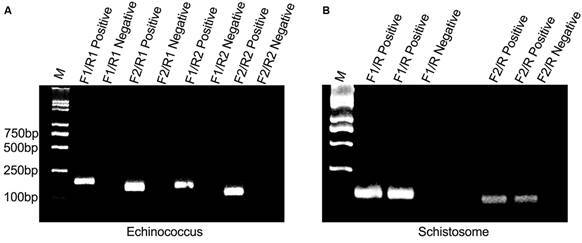
recombinase polymerase amplification (RPA) results based on agarose gel electrophoresis. (A) RPA amplification of *Echinococcus*; (B) RPA amplification of schistosome. The marker and samples name were indicated above the gel image. The marker size was shown on the left. Three independent experiments obtained the similar results, one set of results is presented here.

Multiple primers were designed through sequence alignment, and a combination of primers was screened to determine the optimal set of primers for the final primer sequence. For *Echinococcus*, two forward primers and two reverse primers were designed. These primers were paired in all possible combinations, resulting in four combinations for RPA reactions: F1/R1 (fragment size: 218bp), F1/R2 (fragment size: 165bp), F2/R1 (fragment size: 159bp), and F2/R2 (amplified fragment size: 106bp). For schistosome, two forward primers and one reverse primer were designed. These primers were paired in two combinations for RPA reactions: F1/R (amplified fragment size: 104bp) and F2/R (amplified fragment size: 94bp). The experimental results are shown in Fig. 1. Due to the relatively low sensitivity of agarose gel electrophoresis, plasmids containing the target gene sequence with 10^9^ copies were used as templates to assess the effectiveness of primer design. The amplification results indicated that all primer combinations successfully amplified the corresponding target bands (Fig. 1).

**Fig. 2:**
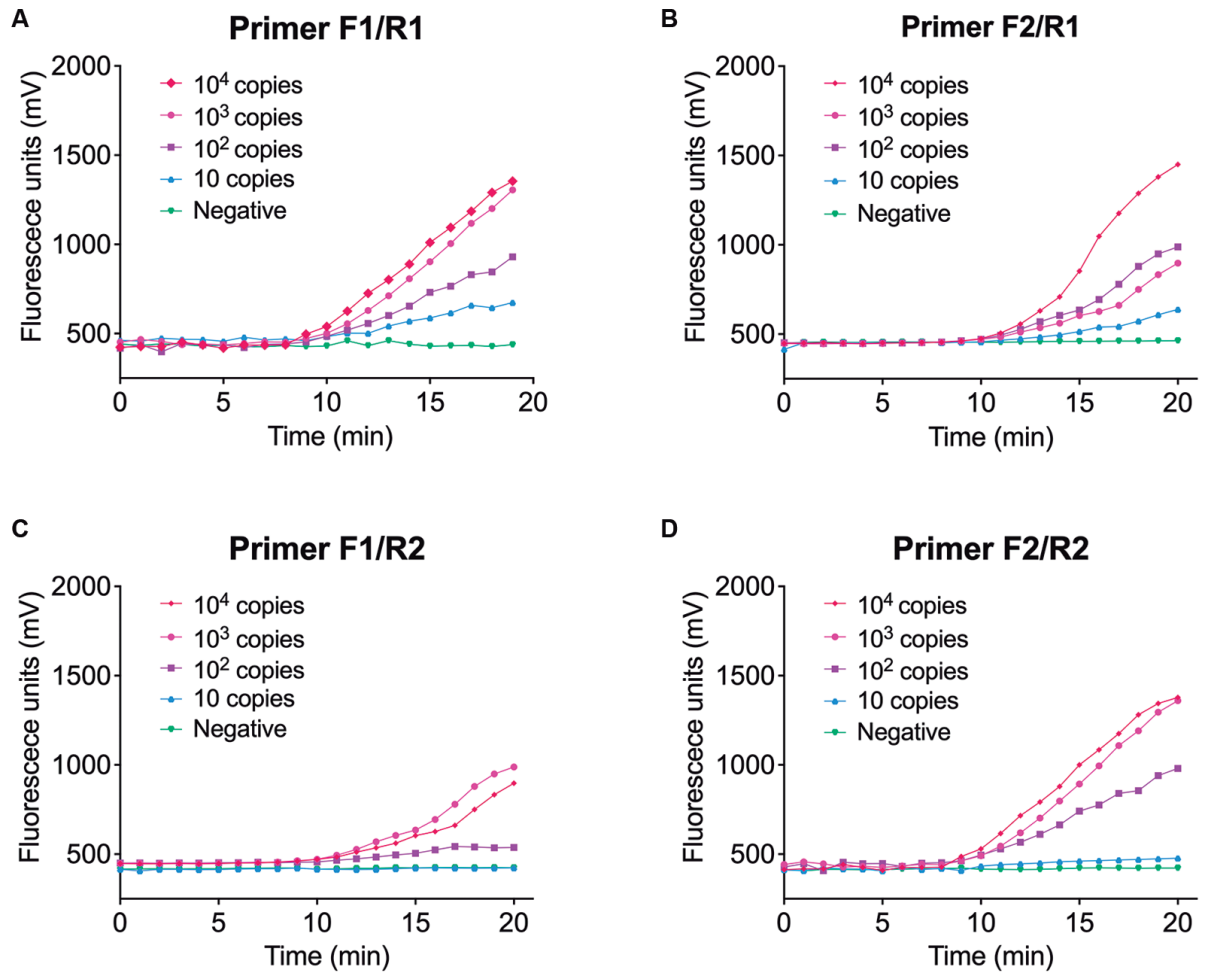
screen of combinations of probes and primers using different concentrations of positive plasmids for *Echinococcus*. (A) Combinations of primer F1 and R1. (B) Combinations of primer F2 and R1. (C) Combinations of primer F1 and R2. (D) Combinations of primer F2 and R2. Each point represents an individual sample. Ten independent experiments obtained the similar results, one set of results is presented here.

**Fig. 3:**
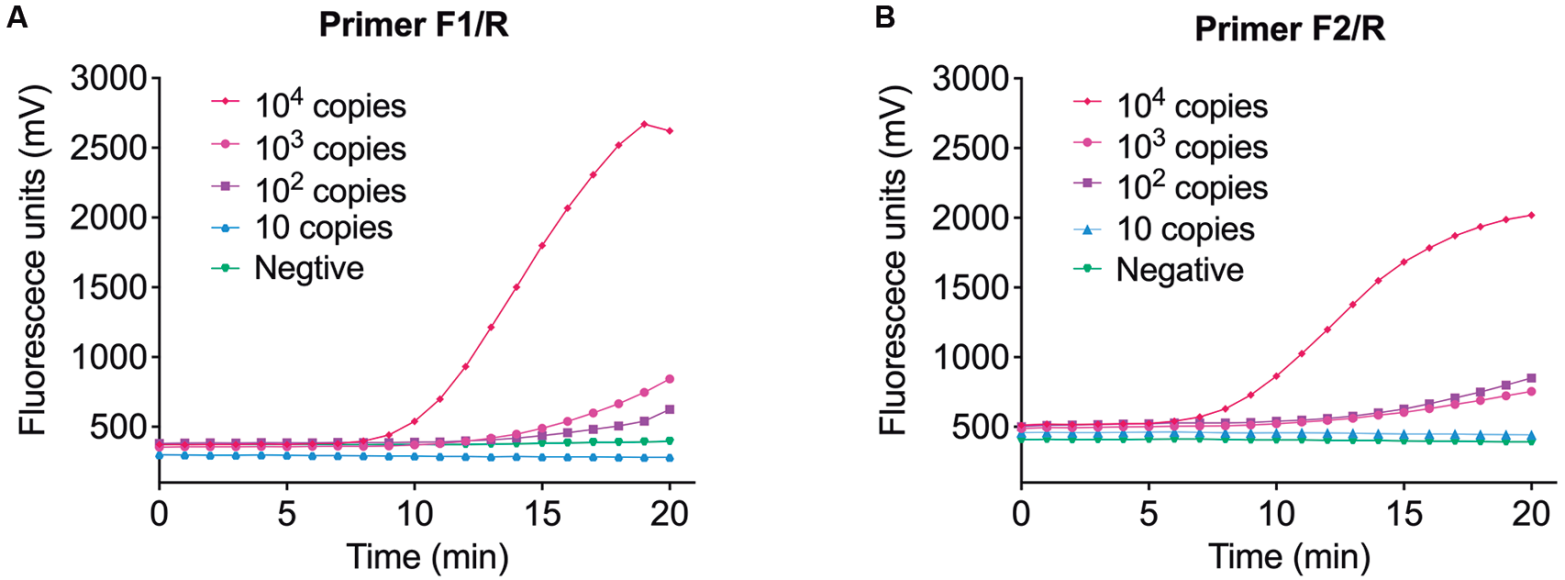
screen of combinations of probes and primers using different concentrations of positive plasmids for schistosome. (A) Combinations of primer F1 and R. (B) Combinations of primer F2 and R. Each point represents an individual sample. Ten independent experiments obtained the similar results, one set of results is presented here.

After the addition of fluorescent probes, different combinations of probes and primers were screened using positive plasmids with concentrations of 10^4^ copies/μL, 10^3^ copies/μL, 10^2^ copies/μL, and 10 copies/μL. The evaluation criteria included the fluorescence values and the limit of detection. For *Echinococcus*, as shown in Fig. 2, primer pairs F1/R1 and F2/R1 successfully amplified down to 10 copies, making them suitable candidate primer-probe combinations. Considering that the fluorescence value of F2/R1 was slightly higher than that of F1/R1, the F2/R1 primer combination was ultimately chosen. For schistosome, as depicted in Fig. 3, primer pairs F1/R and F2/R successfully amplified down to 100 copies, making them suitable candidate primer-probe combinations. Considering that the fluorescence value of F1/R was higher than that of F2/R, the F1/R primer combination was ultimately chosen. The specific primer and probe sequences are provided in Table II.

**TABLE II:** The specific primer and probe sequences for recombinase polymerase amplification (RPA) reactions

Name	Primer sequence
*Echinococcus*	
Forward-primer	5’- TGTTGATTTTGCCTGGATTTGGTATAATTAGTCAT -3’
Reverse-primer	5’- GAAACAACCCATCACAAAACCGGATCACTAACA -3’
Probe	5’- GTGTTTGGGTAGCAGGGTTTGGGGTCATCATATGTTTACTGTTGGGTTG-3’
Schistosome	
Forward-primer	5’-TTTCCAATGATCTTCCGACATGTTA-3’
Reverse-primer	5’-TAGAGTTGTTATTGATAAACAATTCA-3’
Probe	5’-CCGAAGAAACTGGCCTATTGTTCTAAGTTTATTTCATAAGTT-3’

**Fig. 4:**
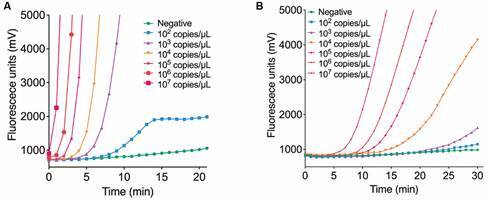
sensitivity examination for (A) *Echinococcus* and (B) schistosome with seven gradients. Overall recombinase polymerase amplification (RPA) system has achieved apparent sensitivity. Each point represents an individual sample. Ten independent experiments obtained the similar results, one set of results is presented here.

We next determined whether the plasmid sensitivity with RPA. Plasmid fragments of *Echinococcus* and schistosome tissue DNA samples were serially diluted tenfold in DEPC water across seven gradients. RPA was used to test the plasmid sensitivity. The results are shown in Fig. 4.

The established RPA system for echinococcosis can detect plasmids at a concentration as low as 10^2^ copies/μL, and within 10 min, it can significantly detect plasmids at a concentration of 10^3^ copies/μL. Simultaneously, the RPA system for schistosomiasis can detect plasmids at a concentration of 10^2^ copies/μL, it can significantly detect plasmids at a concentration of 10^4^ copies/μL within 30 min. The overall RPA system has achieved apparent sensitivity. It is noted that the plasmid sensitivity shown here using RPA is intended to obtain the minimum detectable concentration for on-site rapid detection, which is a different approach from the analytical sensitivity which is often referred to as the limit of detection (LOD).^18^

RPA is a new nucleic acid isothermal amplification technology developed by Piepenburg et al. using protein recombination and repair involved in cell DNA synthesis.^19^ Compared with traditional PCR, which requires instruments for cyclic amplification, the RPA method only needs two primers to realise a reaction,^20^ and can amplify a small amount of template to a detectable level within 3-10 min at an optimal reaction temperature of 37-42ºC. It has been widely used to amplify a variety of different targets, including RNA, miRNA, ssDNA and dsDNA of bacteria, fungi, viruses, protozoa, etc.^15^ It has the advantages of high reaction sensitivity, strong specificity, strong sample tolerance, low dependence on instruments and the ability to integrate multiple detection modes.^21^ At the same time, because RPA is simple to operate under constant temperature conditions, it can reduce the user’s instrument cost. So it is particularly suitable for grassroots and on-site instant testing and can be widely used in in vitro diagnosis, veterinary medicine, food safety, biosafety, agriculture, customs entry and exit disease prevention and control and other fields. And in most cases, the RPA on-site rapid detection method developed in this research makes a person without professional training can complete sample collection, sample preparation, experimental testing and obtain results within half an hour.

*In conclusions* - This study established a rapid detection method for *Echinococcus* and Japanese schistosome samples using the RPA technique. The method allows for the detection of *Echinococcus* and Japanese schistosome presence within 20 min, with an apparent sensitivity of 100 copies/μL, meeting the requirements for on-site rapid detection. The methods developed in this study for detecting these parasites can detect *Echinococcus* or schistosome eggs on-site. These methods are intended for application in parasitic disease monitoring during outbreaks, screening in endemic areas for *Echinococcus* and schistosome, and monitoring incoming individuals from endemic areas at border checkpoints. Furthermore, the research outcomes can be applied to the early diagnosis of these two chronic parasitic diseases, facilitating rapid detection and parasitic disease prevention and control efforts for *Echinococcus* and Japanese schistosome. Meanwhile, more accurate and specific test data still need to be confirmed through further clinical tests.
